# Machine Learning-Based Classification of Retinitis Pigmentosa from Color Fundus Images: A Reproducible Benchmark and Screening-Oriented Pipeline

**DOI:** 10.3390/vision10030055

**Published:** 2026-08-18

**Authors:** Francesco Cappellani, Giovanni Rubegni, Andrea Caruso, Alessia Cosentino, Roberta Torrisi, Grazia Pia Raciti, Marco Mastroeni, Gabriella Lupo, Caterina Gagliano, Massimiliano Salfi

**Affiliations:** 1 Department of Ophthalmology, University of Catania, 95131 Catania, Italy; 2 Department of Medicine and Surgery, University of Enna “Kore”, 94100 Enna, Italy; caterina.gagliano@unikore.it; 3 Ophthalmology Unit, Department of Medicine, Surgery and Neurosciences, University of Siena, 53100 Siena, Italy; 4 vEyes NPO, 95010 Milo, Italy; 5 Department of Electrical, Electronic and Computer Engineering, University of Catania, 95131 Catania, Italy; 6 Department of Biomedical and Biotechnological Sciences, University of Catania, 95131 Catania, Italy

**Keywords:** retinitis pigmentosa, artificial intelligence, machine learning, convolutional neural networks, fundus images, screening

## Abstract

Retinitis pigmentosa (RP) is a rare inherited retinal disorder in which fundus changes may be subtle and heterogeneous, limiting detection from color fundus images. This study evaluated multiple machine learning architectures for binary-RP versus healthy-control classification, and developed a reproducible pipeline for research-oriented screening support. Three publicly available fundus datasets were combined, including 248 RP images and 1045 healthy controls. An 80/20 train–test split was used, with targeted data augmentation applied only to RP images in the training set to address class imbalance. ConvNeXt-Tiny, ResNet101V2, EfficientNet-B0, a baseline classifier, and custom shallow convolutional neural networks were compared using accuracy, precision, recall, F1-score, confusion matrices, and ROC/precision–recall analyses. A compact ShallowCNN provided the best sensitivity–performance trade-off. On the fixed image-level test set, Adam with a learning rate of 0.0005 reached 96.51% accuracy, while SGD with a learning rate of 0.001 achieved 98% RP recall, minimizing false negatives. The trained models were exported to ONNX and integrated into a Windows inference tool. The proposed framework provides an open, reproducible benchmark for technical evaluation, although external validation is required before clinical use.

## 1. Introduction

Retinitis pigmentosa (RP) is a group of rare retinal disorders affecting approximately 1 in 4000 individuals worldwide [1,2]. RP may be non-syndromic or syndromic, with early rod dysfunction resulting in night blindness and peripheral field loss, followed by cone degeneration leading to central vision loss [3,4]. Typical fundoscopic findings include bone spicule pigmentation, vessel attenuation, and specific electrophysiological changes [5,6], although early or atypical cases may exhibit a normal appearance [7]. Additional common findings include posterior subcapsular cataracts and macular edema [8,9,10]. Advances in imaging techniques, such as optical coherence tomography (OCT), fundus autofluorescence (FAF), and electroretinography (ERG), have significantly improved the clinical assessment and phenotypic characterization of RP [11,12].

Despite these advances, identifying RP reliably in routine clinical practice can be challenging because early signs are often subtle, patient presentations are heterogeneous, and imaging conditions vary across devices and centers. In this context, machine learning (ML) applied to color fundus images represents a promising approach to support research-oriented screening and to assist clinicians in flagging cases that warrant further evaluation. Prior work has demonstrated that convolutional neural networks can detect multiple retinal diseases from fundus photographs with high performance when trained on large, well-annotated datasets [13]. Systematic reviews further document broad application of ML techniques to fundus image analysis, evaluating practical trade-offs between model complexity, dataset size, and deployment constraints [14,15]. Recent comprehensive analyses emphasize that model selection must account for intended use case, dataset characteristics, and computational requirements for real-world use [16].

Given these considerations, we designed a comparative study that evaluates both complex pretrained deep architectures and simpler custom shallow networks for the binary classification of RP versus healthy controls using color fundus images. Specifically, we assessed whether highly parameterized pretrained networks outperform lighter architectures on fundus datasets, or whether smaller networks can deliver comparable performance with lower risk of overfitting and reduced computational cost. The model set comprised a baseline fully connected binary classifier, ConvNeXt-Tiny, ResNet101V2, EfficientNet-B0, and two custom shallow convolutional neural networks (CNNs), with and without batch normalization/dropout. All models were trained within a reproducible pipeline using an 80/20 train/test split, with targeted augmentation of RP images to mitigate class imbalance. Hyperparameter tuning included comparisons between Adam and stochastic gradient descent (SGD) optimizers and several learning rates (LRs). Performance was measured using accuracy, precision, recall, F1-score, and confusion matrices, along with receiver operating characteristic (ROC) and precision–recall (PR) curves. The best models were exported and packaged into a Windows-based inference tool for offline research-oriented screening. All code, trained models and experimental notebooks, along with the inference tool, are publicly available through a GitHub repository [17] and a complementary Google Drive (Google LLC, Mountain View, CA, USA) archive [18].

The main contribution of this study is a reproducible multi-source comparison of pretrained and lightweight custom architectures, complemented by sensitivity-oriented evaluation and the release of trained ONNX models and a standalone inference tool.

## 2. Materials and Methods

### 2.1. Dataset Description

This study integrates three independent publicly available retinal fundus image datasets, each providing high-quality images and expert annotations relevant to the analysis of RP.

The Retinal Fundus Multi-Disease Image Dataset (RFMiD) includes 3200 color fundus images covering multiple retinal diseases, including RP, age-related macular degeneration, and diabetic retinopathy [19]. Images were acquired using TOPCON 3D OCT-2000 (TOPCON Corporation, Tokyo, Japan), Kowa VX-10α (Kowa Company, Ltd., Tokyo, Japan), and TOPCON TRC-NW300 (TOPCON Corporation, Tokyo, Japan) cameras. The present study included all 10 RP images available in RFMiD, comprising 8 images acquired using the TOPCON 3D OCT-2000 and 2 using the Kowa VX-10α. The 669 healthy control images available in RFMiD were not included in the experimental dataset to avoid further increasing the class imbalance between RP and healthy controls. All images were independently annotated by two expert ophthalmologists with consensus adjudication.

The Color Fundus Images for Eye Disease Detection dataset comprises 5335 fundus and anterior segment images collected at Anawara Hamida Eye Hospital and B.N.S.B. Zahurul Haque Eye Hospital, Faridpur, Bangladesh [20]. Images were acquired using TOPCON TRC-50DX (TOPCON Corporation, Tokyo, Japan) and TL-211 (TOPCON Corporation, Tokyo, Japan) cameras . A total of 139 RP fundus images and 1024 healthy control images were selected. Each image received a single diagnostic label assigned by qualified ophthalmologists.

The Retinal Images for Pigment Signs (RIPS) dataset includes 120 color fundus images, of which 99 correspond to RP patients and 21 to healthy controls [21]. These images were acquired using a Canon CR4-45NM camera (Canon Inc., Tokyo, Japan).

**Figure 1:**
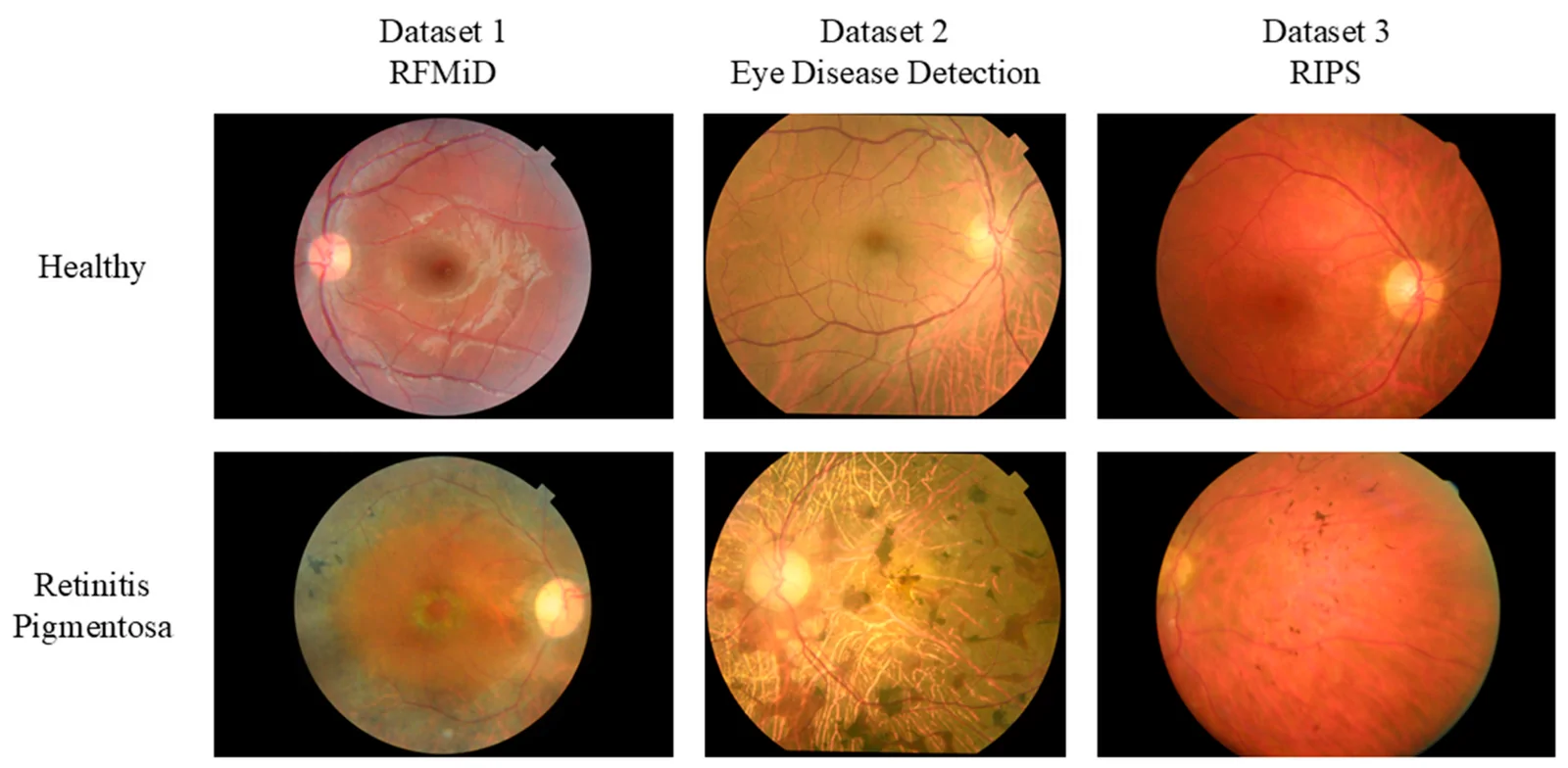
Representative healthy-control and retinitis pigmentosa fundus images from the three source datasets: RFMiD, the Eye Disease Detection dataset, and RIPS. The RFMiD healthy-control image is shown for visual comparison, but this was not included in the experimental dataset.

The final aggregated dataset comprised 248 RP images, including 10 from RFMiD, 139 from the Color Fundus Images for Eye Disease Detection dataset, and 99 from RIPS, together with 1045 healthy control images, including 1024 from the Color Fundus Images for Eye Disease Detection dataset and 21 from RIPS, for a total of 1293 images. Because the released datasets did not provide consistent subject identifiers, the number of unique patients could not be reliably determined, and patient-level stratification was not feasible. The reported evaluation should therefore be interpreted as image-level. Representative healthy and RP fundus images are shown in Figure 1.

### 2.2. Experimental Setup and Evaluation Metrics

All experiments were conducted on Google Colab using an NVIDIA T4 GPU (16 GB), with data being stored on Google Drive. Models were implemented in Python (3.12.12) using PyTorch (2.8.0), performance metrics were computed with Scikit-learn, and visualizations were generated using Matplotlib (3.10.0) and Seaborn (0.13.2). Training monitored accuracy and loss on the training set and a dynamically re-sampled validation set. Final evaluation was performed on an independent test set using test accuracy, a confusion matrix, and a detailed classification report, from which the following metrics were derived:

Accuracy:

$$\mathrm{Accuracy}=\frac{TP+TN}{TP+TN+FP+FN}$$

Precision:

$$\mathrm{Precision}=\frac{TP}{TP+FP}$$

Recall:

$$\mathrm{Recall}=\frac{TP}{TP+FN}$$

F1-score:

$$F1\text{-}\mathrm{Score}=2\cdot \frac{\mathrm{Precision}\cdot \mathrm{Recall}}{\mathrm{Precision}+\mathrm{Recall}}$$

The macro average is defined as:

$$\mathrm{Macro}\;\mathrm{Average}=\frac{1}{C}\sum_{i=1}^{C}M_{i}$$

The weighted average is defined as:

$$\mathrm{Weighted}\;\mathrm{Average}=\frac{1}{N}\cdot \sum_{i=1}^{C}n_{i}\cdot M_{i}$$

where C is the number of classes, Mi is the metric for class i, ni is the number of samples in class i, and N is the total number of samples. For the best-performing network, further analyses included plotting the ROC curve and calculating the area under the curve (AUC), with the true positive rate (TPR) and false positive rate (FPR) defined as:

$$\mathrm{TPR}=\frac{TP}{TP+FN}$$

$$\mathrm{FPR}=\frac{FP}{FP+TN}$$

The PR curve was also examined, providing complementary insight in cases of imbalanced class distributions.

### 2.3. Data Collection and Pre-Processing

The final aggregated dataset included 248 RP images and 1045 healthy controls; this was organized, before model training, into a fixed training set containing 199 RP images and 836 controls, and a fixed test set containing 49 RP images and 209 controls. The same predefined training and test partitions were used for all experiments, and no additional random train–test split was performed during model training or evaluation. The partition maintained an approximately 80/20 distribution within each included source and class: RFMiD contributed 8/2 RP images, the Color Fundus Images dataset contributed 111/28 RP and 819/205 control images, and RIPS contributed 80/19 RP and 17/4 control images to the training/test sets, respectively.

After the fixed training and test sets had been defined, data augmentation was applied on the fly to images in the training directory using horizontal flips, rotations, affine transformations, and mild color adjustments. Test images underwent only resizing, tensor conversion, and normalization, without random augmentation. At each epoch, the predefined training set was randomly repartitioned into 80% training and 20% validation subsets, whereas the independent test set remained fixed and was used only for final evaluation.

### 2.4. Architecture Comparison

The first phase of the study systematically compared several neural network architectures for binary classification of RP versus healthy controls. The comparison prioritized RP sensitivity while also reporting standard metrics for controls to evaluate sensitivity–specificity trade-offs. All models were trained on the aggregated training set and evaluated on the held-out test set.

In detail, the following experiments were conducted:

1. **Experiment 1—Baseline fully connected binary classifier:** a multilayer perceptron with vectorized inputs, three dense layers (128-64-1 neurons), ReLU activations, sigmoid output, binary cross-entropy loss, and Adam (LR = 0.001).
2. **Experiment 2—ConvNeXt-Tiny:** adapted for binary classification by replacing the final head with a single output neuron; trained with binary cross-entropy with logits and Adam (LR = 0.001).
3. **Experiment 3—ShallowCNN:** two convolutional layers with ReLU and max-pooling, followed by a small fully connected head; binary cross-entropy and Adam (LR = 0.001).
4. **Experiment 4—ResNet101V2:** fine-tuned, with the final layer replaced by a single output neuron; binary cross-entropy with logits and Adam (LR = 0.001).
5. **Experiment 5—ResNet101V2-FZ (frozen backbone):** only the classifier layer was trained; AdamW with LR = 0.0001 for frozen weights and 0.001 for the head.
6. **Experiment 6—ShallowCNN-BN:** ShallowCNN with batch normalization and dropout; two convolutional layers (BN + ReLU + max-pooling) followed by fully connected layers; binary cross-entropy and AdamW (LR = 0.001).
7. **Experiment 7—EfficientNet-B0:** final head replaced with a single output neuron; binary cross-entropy with logits and Adam (LR = 0.0005).

Each model was trained up to a predefined maximum number of epochs, with early stopping based on validation loss. Based on test set metrics, ShallowCNN achieved the highest RP recall while maintaining balanced control performance, minimizing false negatives that are critical in screening-oriented contexts. It was therefore selected for further refinement and hyperparameter tuning.

### 2.5. Model Refinement

The ShallowCNN model was further optimized by testing two optimizers (Adam and SGD) and two learning rates (0.001 and 0.0005). Adam adapts parameter-specific learning rates using gradient moments for faster convergence, while SGD uses a fixed rate, offering more-stable but slower updates. Each configuration employed early stopping if validation loss did not improve for five consecutive epochs. This allowed assessment of how optimizer and learning rate influenced the balance of sensitivity and specificity for RP detection.

## 3. Results

### 3.1. Architecture Comparison Results

The evaluated architectures were assessed on the held-out test set using accuracy, precision, recall, and F1-score, as well as macro and weighted averages (Table 1). ShallowCNN achieved balanced performance, with 94.96% accuracy, RP recall of 0.84, and a macro F1-score of 0.92. EfficientNet-B0 had slightly higher macro precision (0.97) but lower RP recall (0.69), indicating more false negatives, while ResNet101V2-FZ showed similar accuracy (93.41%) but lower RP recall (0.73) and macro F1-score (0.88). These results highlight ShallowCNN as the best trade-off between RP sensitivity and overall performance. Given the clinical priority of minimizing false negatives in screening, ShallowCNN was selected for further hyperparameter tuning.

ConvNeXt classified all test images as controls; consequently, its RP precision, recall, and F1-score were all zero, while the weighted F1-score remained 0.73 because of the greater contribution of the majority control class.

### 3.2. Model Refinement Results

Four hyperparameter configurations of ShallowCNN were evaluated, with training and test results summarized in Table 2.

Adam with LR = 0.0005 achieved the best balance, producing the highest test accuracy (96.51%) and strong F1-scores for both classes, indicating balanced performance across the two classes on the fixed test set. Adam with LR = 0.001 converged faster, but with slightly reduced RP detection performance.

SGD configurations favored RP sensitivity: SGD with LR = 0.001 achieved the highest RP recall (98%), minimizing false negatives at the cost of more false positives, while LR = 0.0005 improved overall balance, producing higher macro and weighted F1-scores, and maintaining high RP sensitivity.

**Figure 2:**
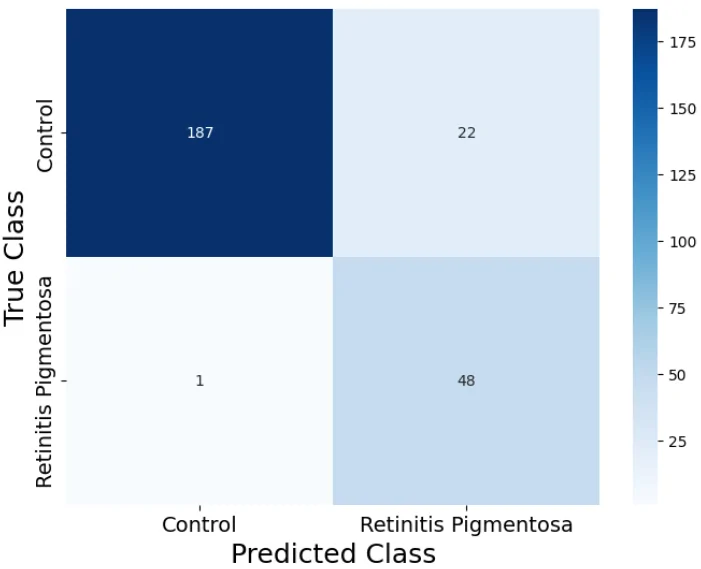
Confusion matrix of the ShallowCNN model trained using the SGD optimizer with LR = 0.001.

**Figure 3:**
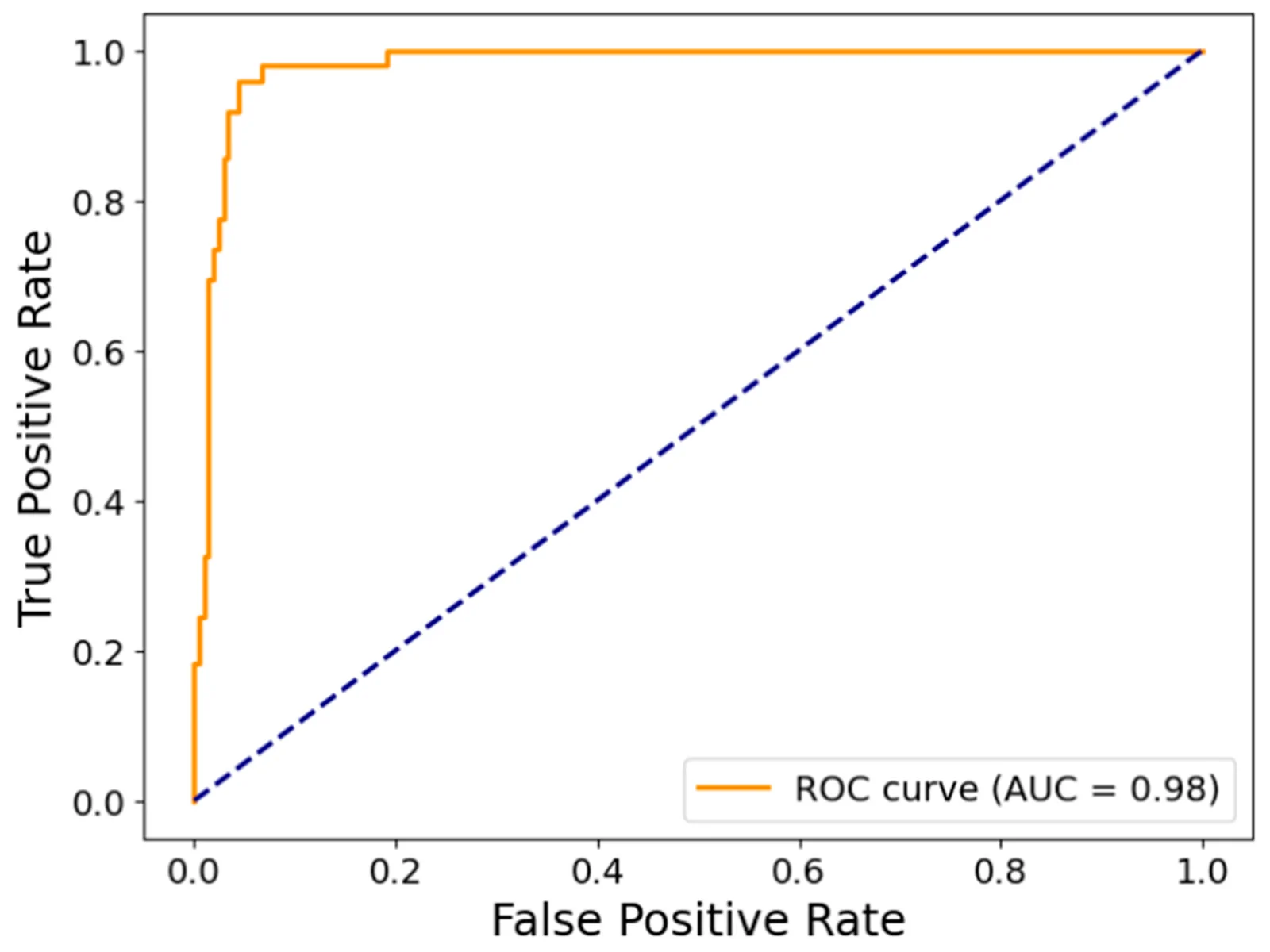
ROC curve of the ShallowCNN model trained using the SGD optimizer with LR = 0.001.

The confusion matrix for SGD with LR = 0.001, which achieved the best performance in terms of reducing RP false negatives, is shown in Figure 2, with its ROC curve being shown in Figure 3. ROC and PR comparisons for all four configurations are provided in the Supplementary Materials.

### 3.3. Inference Tool

The four trained models derived from the selected ShallowCNN were exported for standalone inference and packaged in a simple Windows graphical user interface (GUI) tool [17,18] to facilitate offline evaluation and demonstration. Models were produced in PyTorch .pt format within Google Colab and then converted to Open Neural Network Exchange (.onnx) format using the Python script provided in the repositories [17,18].

The Windows inference tool is implemented in .NET 8.0 to be executed on Windows platforms. The tool’s main capabilities are:

- Selection of any .onnx model provided with the tool, or import of a custom .onnx model, including models retrained from the supplied base models or completely new models.
- Loading of a retinal fundus image, either a healthy control or an RP image.
- Execution of single-image inference that outputs a binary prediction (Healthy/RP) and a model confidence score between 0 and 100, with 0 representing a healthy prediction with maximum confidence, 100 representing an RP prediction with maximum confidence, and scores near 50 representing lower confidence.

**Figure 4:**
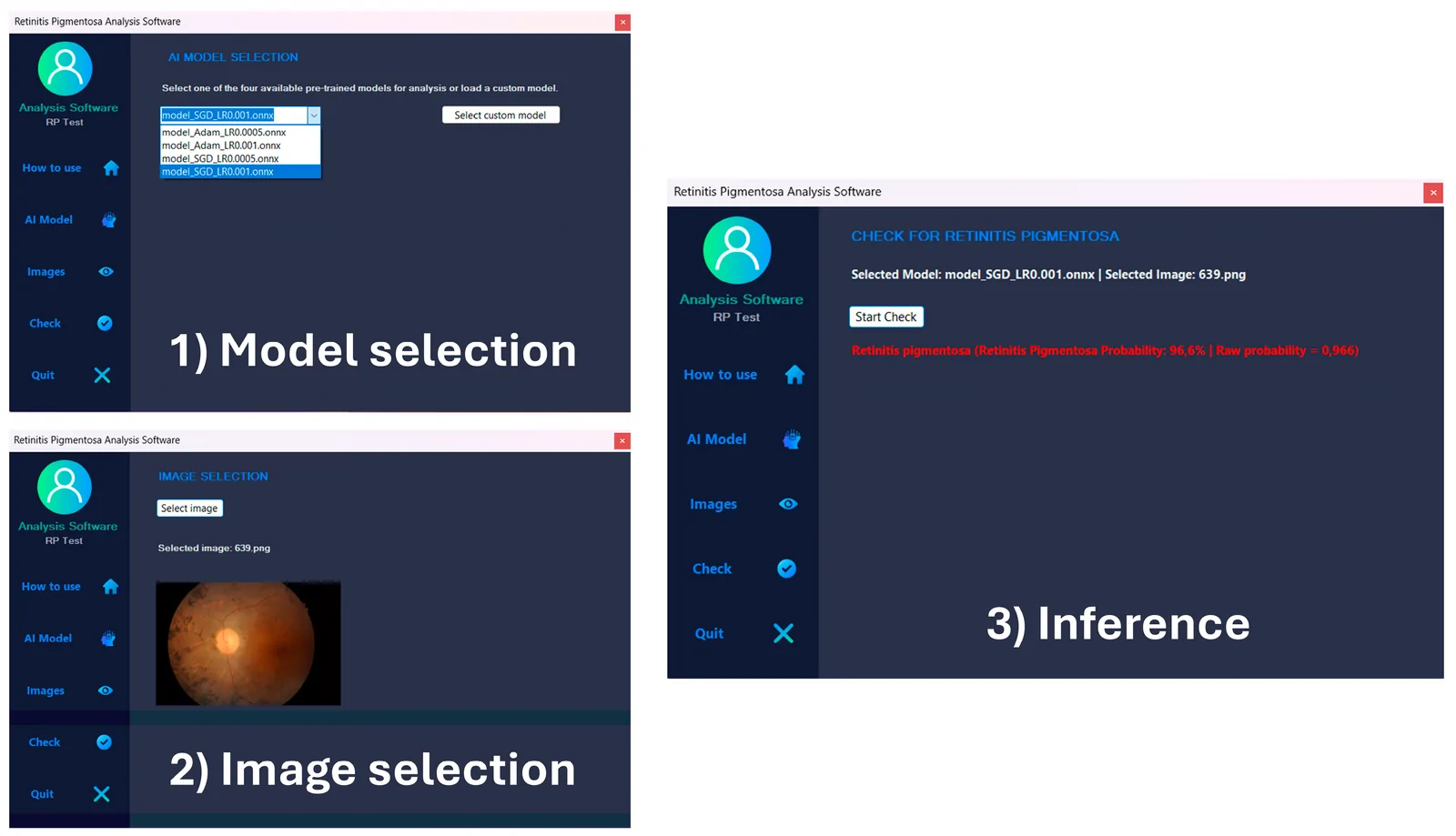
Windows inference tool workflow: an .onnx model is selected, a fundus image is chosen, and the software shows the inference result with predicted class and confidence.

The GUI is intentionally lightweight and intended for research demonstration; it does not constitute a validated clinical device. Example screenshots are provided in Figure 4.

## 4. Discussion

This study systematically compared multiple machine learning architectures for binary classification of RP versus healthy controls using heterogeneous publicly available fundus datasets. Despite the inclusion of large pretrained models, a compact ShallowCNN achieved the best balance between RP sensitivity and overall performance, suggesting that, with limited disease samples and heterogeneous acquisitions, higher model complexity does not guarantee superior results. Lighter architectures may offer greater robustness and lower overfitting risk in this setting.

Refinement of ShallowCNN showed that optimization strategy strongly affects the sensitivity–specificity trade-off. Adam favored faster convergence and higher overall accuracy, whereas SGD increased RP recall at the cost of more false positives. In screening contexts, prioritizing sensitivity to minimize missed RP cases is advisable, with false positives addressed clinically during subsequent evaluation.

The focus on RP sensitivity aligns with prior work. Masumoto et al. showed high sensitivity and specificity in RP detection using ultra-widefield pseudo-color and autofluorescence images [22]. Chen et al. investigated AI-assisted RP detection from color fundus photographs using transfer learning architectures and model interpretability analysis [23], while more recent work explored deep learning classification of RP inheritance forms using synthetic data expansion in a rare-disease setting [24].

Due to RP rarity and class imbalance, PR analysis complements ROC evaluation; Lee et al. highlighted PR utility in imbalanced ophthalmic datasets [25], and Miere et al. reported high PR-AUC in fundus autofluorescence classifiers for inherited retinal diseases, including RP [26]. This study’s combined use of ROC and PR curves follows these recommendations.

Targeted augmentation of RP images stabilized minority-class performance, consistent with evidence that augmentation and imbalance-aware training improve generalization without synthetic data [27]. Geometric and mild photometric transformations preserved clinically plausible retinal structures while increasing realistic variability.

Although this work focused on binary RP versus control classification, machine learning can capture richer disease information. Nagasato et al. demonstrated visual function estimation from ultra-widefield RP images [28], and multimodal approaches combining fundus photography, autofluorescence, and OCT have been explored for inherited retinal diseases [26,29], suggesting potential extensions to prognostic or multimodal applications.

Future extensions may also include broader comparisons with architectures such as VGG19, DenseNet169, ResNet152, and EfficientNet-B4, together with heatmap-based interpretability analyses to examine image regions contributing to model predictions.

Limitations include a lack of patient-level stratification; this may have influenced reported performance estimates if multiple images from the same subject occurred in different partitions. Moreover, differences in acquisition devices and class distributions across the source datasets may have introduced source-related shortcut learning, and limited cross-dataset generalizability [30]. The use of a single fixed split, including 49 RP test images, also limits assessment of performance variability across alternative data partitions. Future work should include repeated or cross-validation experiments, preferably using patient-level separation, as well as per-dataset and leave-one-dataset-out evaluations to assess cross-source generalization. While test set results are promising, external multicenter validation and prospective studies are required prior to clinical application. The Windows inference tool should therefore be considered a research and technical evaluation resource, not a validated clinical decision-support device.

## 5. Conclusions

This study evaluated different convolutional architectures for the binary classification of RP from color fundus images. A compact shallow convolutional neural network trained on aggregated public fundus datasets showed promising accuracy and RP recall on a fixed image-level test set. These results indicate that lightweight architectures can be effective for rare retinal disease detection when data are limited and heterogeneous. Optimization strategy allows tuning of the sensitivity–specificity balance according to research or screening priorities. Although external validation and explicit handling of domain shift are required before clinical translation, the proposed framework provides a reproducible benchmark and practical tools to support further investigation and methodological development.

## Figures and Tables

**Table 1:** Training epochs (N) and class-specific metrics computed on the same fixed test set of 209 controls and 49 RP images, with RP considered the positive class. A: accuracy; P: precision; R: recall; F1: F1-score; C: control; MP: macro precision; MR: macro recall; MF1: macro F1-score; WF1: weighted F1-score.

ID	NAME	N	A (%)	P (C)	P (RP)	R (C)	R (RP)	F1 (C)	F1 (RP)	MP	MR	MF1	WF1
1	BinaryClassifier	10	92.97	0.94	0.87	0.98	0.72	0.96	0.79	0.91	0.85	0.87	0.93
2	ConvNext	7	81.01	0.81	0.00	1.00	0.00	0.90	0.00	0.41	0.50	0.45	0.73
3	ShallowCNN	10	94.96	0.96	0.89	0.98	0.84	0.97	0.86	0.93	0.91	0.92	0.95
4	ResNet101V2	6	89.15	0.88	1.00	1.00	0.43	0.94	0.60	0.94	0.71	0.77	0.87
5	ResNet101V2-FZ	20	93.41	0.94	0.90	0.98	0.73	0.96	0.81	0.92	0.86	0.88	0.93
6	ShallowCNN-BN	15	82.95	0.83	0.86	1.00	0.12	0.90	0.21	0.84	0.56	0.56	0.77
7	EfficientNetB0	15	94.19	0.93	1.00	1.00	0.69	0.97	0.82	0.97	0.85	0.89	0.94

**Table 2:** Training and test-set results for the selected ShallowCNN model under different hyperparameter settings. B denotes the epoch with the highest observed validation accuracy among N training epochs; the validation subset was randomly re-sampled at each epoch. TL, TA, VL, and VA refer to the selected epoch. All test metrics were computed on the same fixed test set of 209 controls and 49 RP images, with RP considered the positive class. A: accuracy; P: precision; R: recall; F1: F1-score; C: control; MP: macro precision; MR: macro recall; MF1: macro F1-score; WF1: weighted F1-score.

Variant	N	B	TL	TA (%)	VL	VA (%)	A (%)	P (C)	P (RP)	R (C)	R (RP)	F1 (C)	F1 (RP)	MP	MR	MF1	WF1
Adam, LR: 0.001	16	11	0.09	96.63	0.05	99.08	94.96	0.99	0.82	0.95	0.94	0.97	0.88	0.90	0.95	0.92	0.95
Adam, LR: 0.0005	13	8	0.09	96.94	0.04	98.47	96.51	0.98	0.90	0.98	0.92	0.98	0.91	0.94	0.95	0.94	0.97
SGD, LR: 0.001	19	14	0.17	94.26	0.09	96.32	91.09	0.99	0.69	0.89	0.98	0.94	0.81	0.84	0.94	0.87	0.92
SGD, LR: 0.0005	18	13	0.21	93.34	0.16	96.01	93.80	0.99	0.77	0.93	0.96	0.96	0.85	0.88	0.95	0.91	0.94

## Data Availability

The data presented in this study were derived from the publicly available datasets cited in the manuscript. Source code, trained models, supplementary figures, and the inference tool are available in the GitHub repository and Google Drive archive listed in References [17,18]. Further inquiries can be directed to the corresponding author.

## Supplementary Materials

The following supporting information can be downloaded at: https://github.com/vEyesLab/RPvsControl and https://drive.google.com/drive/folders/1h8JBL9VFNns4xiifs3-8QyxCR--Wv9es (accessed on 7 May 2026), Supplementary data, images, source code, exported CNN models, and the standalone inference tool are available through the GitHub repository and complementary Google Drive archive cited in the References [17,18].

## Abbreviations

AI	artificial intelligence
AUC	area under the curve
CNN	convolutional neural network
ERG	Electroretinography
FAF	fundus autofluorescence
GUI	graphical user interface
LR	learning rate
ML	machine learning
ONNX	Open Neural Network Exchange
OCT	optical coherence tomography
PR	Precision–recall
RFMiD	Retinal Fundus Multi-Disease Image Dataset
RIPS	Retinal Images for Pigment Signs
ROC	receiver operating characteristic
RP	retinitis pigmentosa
SGD	stochastic gradient descent
